# Impact of increasing the relative availability of meat-free options on food selection: two natural field experiments and an online randomised trial

**DOI:** 10.1186/s12966-021-01239-z

**Published:** 2022-01-31

**Authors:** Rachel Pechey, Paul Bateman, Brian Cook, Susan A. Jebb

**Affiliations:** University of Oxford, Radcliffe Primary Care Building, Radcliffe Observatory Quarter, Woodstock Road, Oxford, OX2 6GG UK

**Keywords:** Meat, Availability, Vegetarian, Purchasing

## Abstract

**Background:**

Increasing the availability of lower energy-density foods is a promising intervention to encourage healthier food purchasing but few studies have examined the effect of increasing availability of meat-free meals to promote more sustainable purchasing. We report three studies, all examining the impact of altering the availability of meat-free meals on meal selection.

**Methods:**

Study 1 (a natural experiment in one university cafeteria) examined the impact of altering the ratio of meat-free meals (one meat-free and two meat, to two meat-free and one meat) on weekly sales of meals containing meat. Study 2 (a natural experiment in 18 worksite cafeterias) examined the impact on meat-free meal sales of a menu change designed to increase the availability of meat-free meals. Study 3 (an online study of 2205 UK-representative adults) compared meal selections when participants were randomised to ranges comprised of (a) one meat-free, three meat options; (b) two meat-free, two meat; or (c) three meat-free, one meat.

**Results:**

Study 1 suggested a significant decrease in the proportion of sales of meat options when the availability of meat-free options increased (− 19.9 percentage points; 95%CIs:-25.2,-14.6), with no evidence of changes to meat-based meal sales in other university cafeterias during the same period. Findings from Study 2 were mixed: multilevel regressions found no evidence of an increase in meat-free meals following the menu change (2.3 percentage points; 95%CIs: − 1.3,5.9), while interrupted time-series analyses suggested sales did increase (2.3; 95%CIs: 0.4,4.2), but implementation of the planned change was limited. In Study 3 reducing meat-free options from 50 to 25% reduced participants’ selection of meat-free options (odds ratio 0.35; 95%CIs: 0.26,0.46), while increasing meat-free options from 50 to 75% increased meat-free selections (odds ratio 2.43; 95%CIs: 1.94,3.04). There was no evidence effects were moderated by gender, socioeconomic status or usual meat consumption.

**Conclusion:**

Increasing the availability of meat-free options is effective at reducing meat selection and purchasing for different ratios of meat to meat-free options. The magnitude of the effect is uncertain, but with no evidence of differences in response by demographic groups when directly tested.

**Trial registration:**

Study 3: Open Science Framework; https://osf.io/ze9c6; 6/8/2020.

**Supplementary Information:**

The online version contains supplementary material available at 10.1186/s12966-021-01239-z.

## Background

The urgent need to promote more sustainable consumption to protect planetary health is increasingly recognised [1, 2]. Changing diets, especially reducing meat and dairy consumption [3], could lead to substantial benefits both in terms of health and environmental impact [2]. Higher red and processed meat consumption is a significant risk factor for non-communicable diseases [4] – the leading cause of death worldwide [5] and meat is the largest food contributor to greenhouse gas emissions [6].

One promising intervention is to alter the availability of meat vs. meat-free meals. A recent Cochrane review found that altering the availability of a particular set of food options changes their selection [7], albeit with low overall certainty. However, no evidence was identified in this review targeting the availability of meat-free options. Similarly, a systematic review of the impact of interventions that aim to restructure physical micro-environments on selection of meat products did not identify any studies relating to increasing the availability of vegetarian food to reduce meat consumption [8]. Since then, an experimental field study in one student cafeteria has suggested that increasing the percentage of vegetarian meals from 25 to 50% leads to an approximately 40% rise in vegetarian meal sales, with observational data from two further university cafeterias also showing increased meat-free meal availability was associated with increased meat-free purchasing [9].

Here we report the findings of three studies examining the impact of altering the availability of meat-free meals on meal selection. First, an evaluation of a natural experiment in a single university cafeteria over one university term. Second, a test of the impact of availability in a broader context, exploring the impact of a catering provider changing their menus to include more meat-free options in 18 worksite cafeterias across the UK. Third, a large experimental study conducted online to investigate whether the impact of meat-free availability on the selection of meat-free meals varies by participant characteristics.

## Methods

### Study 1: natural experiment in a university cafeteria

This study in one university cafeteria aimed to estimate the effect on sales of meat-based hot meals after the relative availability of meat-free hot meals was increased over a 4-month period. The analysis plan was pre-registered on the Open Science Framework (https://osf.io/w26hc).

#### Setting

One nationwide foodservice provider operates multiple cafeterias across the University of Oxford. The cafeterias cater to staff, students and visitors to the university. There are usually alternative venues nearby, but the university cafeterias are often closest, being part of university buildings. A choice of hot meals (served by cafeteria staff) or pre-packaged cold items are available at each cafeteria.

Between 23 September 2019 and 10 January 2020, the catering provider changed their menu at one of these cafeterias.

#### Intervention

The cafeteria menu included three main meals that are offered 5 days per week, following a 3-week menu cycle. At baseline (25 April – 22 September 2019), two meat meals and one meat-free option were available daily (meat includes red or white meat and fish). Between 23 September 2019 and 10 January 2020, this ratio was shifted to one meat meal and two meat-free meals each day. Meals were priced according to the usual pricing strategy, with meat-free meals standardly being cheaper than meat meals.

The intervention period was taken to be between 23 September and 13 December (12 weeks), excluding sales in late December and January, due to concerns about differences in sales over the Christmas period and during January (due to Veganuary).

#### Data

Sales data (number of units sold) for the site for all products over the baseline and intervention periods were obtained. These data were collected from the site’s electronic point-of-sale tills (cash registers).

##### Comparison sites

Data were also obtained for the same period from 11 other University of Oxford cafeteria sites operated by the same caterer, where no changes had been made to the availability of meat-free meals. Sites varied in the number of meal options they offered, but selected the meals to offer from the same base menu.

Weekends were excluded as some sites were closed at weekends, and among those open the meals offered changed, and did not always follow the same pattern of meat vs. meat-free availability.

#### Analyses

##### Primary analysis

An interrupted time series predicted the percentage of hot meals containing meat purchased per week from the intervention cafeteria, depending on the relative availability of meat-free meals (modelled using a dummy variable). Covariates included a dummy variable indicating whether it was during university term (vs. holidays).

The above analysis was then repeated on the data from each of the other University of Oxford cafeterias. Inference criteria were set such that the primary analysis (in the intervention cafeteria) must be significant at *p* < 0.0045 (i.e. adjusting the *p*-value using a Bonferroni correction accounting for 11 control sites), and none of the results from the other cafeterias significant at *p* < 0.0045.

An additional sensitivity analysis was conducted predicting the percentage of hot meals containing meat purchased daily, rather than weekly, from the intervention cafeteria. This included day of the week as an additional covariate.

##### Secondary analyses

These included investigating possible compensatory effects, by examining the effect on sales of all lunchtime options containing meat. In addition, to ensure that customers were not put off by the changes to the menu, the effect on total sales was examined. (A fourth aim, included in the pre-registration, examining the environmental impact of changes, was not investigated due to data on meal ingredients being unavailable.) These analyses were conducted just on lunchtime sales at the intervention cafeteria site, to further explore effects identified in the primary analysis, rather than to provide evidence of an effect. Inference criteria were set at *p* < 0.016, to adjust for the multiple analyses using the intervention cafeteria data.

To examine compensatory effects, an interrupted time series predicted the percentage of all lunchtime options containing meat (hot meals, sandwiches, panini, jacket potatoes) purchased per week. For overall sales, the analysis was similar, but predicting the number of all lunchtime options purchased per week.

### Study 2: natural experiment in worksite cafeterias

The primary research question for this study was whether the proportion of meat-free meal purchases increased following a planned change by a UK foodservice provider to increase the proportion of meat-free meals on the menu. This study analysed sales data for the 8 weeks before and 8 weeks following this menu change. The analysis plan was pre-registered on the Open Science Framework (https://osf.io/36yfx/).

#### Intervention

From 7th September 2020, the catering provider changed their menus to (1) introduce meat-free Mondays; (2) increase their range of meat-free meals. While the catering provider designs the menus, chefs at each site can choose options off the menus to prepare each day (e.g. preparing 2 out of the 4 suggested options on the menu).

#### Data

Data from electronic point-of-sale tills were provided by the catering provider. Study data comprised the purchases of each meal option for sites open for the 8 weeks before and 8 weeks following the menu change.

Eighteen sites, which sold more than 100 meals per week, were included in these analyses. Inclusion criteria based on sales were used given the greater likelihood of both higher sales variation and running reduced menu options in smaller sites, potentially exacerbated by the Covid-19 pandemic.

#### Setting

The 18 sites, based across England, were predominantly distribution centres/warehouses (10 sites) or manufacturing sites (7 sites), with one site being an office.

#### Analysis

The primary outcome was the proportion of meat-free meals purchased per week in each cafeteria. Meals where it could not be identified whether these were meat-free or meat-based (e.g. Main Meal A) were excluded from analyses. ‘Meat-based’ included meals containing any meat or fish.

Two analyses were used to investigate the primary research question. First, multilevel linear regression of the proportion of meat-free meals purchased per week was conducted, with random effects for site, comparing sales before and after the menu change (modelled using a dummy variable). A continuous variable indicating week was included to explore possible time trends, and the baseline proportion meat-free meals (on offer in the 4 weeks prior to the study period) was also added to models. Weeks with zero meat-free meals offered at a site were treated as missing data in multilevel models, given that if no meat-free meals were offered during a particular time period, these could not have been purchased. (No sites offered zero meat meals in a given week). Sites with at least 4 weeks’ data that did not include zero meat-free meals offered, both before and after the menu change (i.e., at least 8 weeks in total) were included in analyses. However, excluding observations where no meat-free meals were offered could curtail our estimates of impact – particularly as this was targeted in the menu change being assessed. Second, therefore, an interrupted time-series analysis was conducted on weekly data aggregated across sites, allowing us to include these zero values for individual sites. These analyses were run on sites with data for all 16 weeks (8 weeks before and eight after the menu change). Lags of 4 were selected, given the 4-weekly menu cycle used within sites.

As chefs at each site have discretion over which meals to select from the menus, a secondary aim was to examine whether the proportion of meat-free meals offered increased following the menu change. Analyses of the secondary outcome (the proportion of meat-free meals offered per week in each cafeteria) followed the same methods. Offered is defined as being sold at least once in a cafeteria during each week – we were not able to identify meals that were offered but had zero sales.

An additional (not pre-registered) sensitivity analysis was also conducted, exploring the impact of the menu change for those sites where the proportion of meat-free meals offered per week had increased by at least one percentage point following the menu change, using multilevel linear regression.

### Study 3: online experiment

Online studies have been used in the context of manipulating the availability of healthier (vs. less-healthy) foods and non-alcoholic (vs. alcoholic) drinks [10, 11], but not (to our knowledge) for exploring altering the availability of meat vs. meat-free options.

The aims of Study 3 were to estimate the impact of altering the relative availability of meat vs. meat-free food upon the selection of meat-free food options, and to explore the impact of participant characteristics – in particular, baseline consumption of meat-based main meals, socioeconomic status and gender – on the effect of altering availability of meat vs. meat-free options on selection of a meat-free main meal option.

#### Participants

A UK sample of 2205 adults was recruited from a global market research agency panel (Dynata), with quotas set by highest educational qualification (to achieve equal numbers in higher vs. lower groups), and to achieve a representative sample of the UK population by age and gender. Individuals who had dietary restrictions (e.g. vegetarians) were excluded to ensure that participants felt they had a choice between the options offered.

The sample size was calculated assuming around 25% of selections would be meat-free in the reference condition with a small effect size for the effect of altering meat-free availability (increase with an odds ratio of 1.5; decrease with an odds ratio of 0.66). For power of 0.9 (alpha =0.05), the sample size per group to detect an increase (odds ratio of 1.5) was calculated to be 624, and to detect a decrease (odds ratio of 0.66) was 729. Allowing 735 for each of the three groups (in case of missing data) gave a total sample size of 2205.

#### Design

This was a between-subjects, online study, comparing choices between main meal options from ranges comprised of (a) one meat-free, three meat options; (b) two meat-free, two meat; (c) three meat-free, one meat options [based on the standard number of options in observed in cafeteria offerings previously], run in August and September 2020.

The study was pre-registered on the Open Science Framework (registration: https://osf.io/ze9c6). The project was reviewed by, and received ethics approval through, the University of Oxford Central University Research Ethics Committee [R70304/RE001].

#### Measures and materials

##### Food options

Eight options were identified for the main meals: four meat-free (three bean chilli; veggie burger; cauliflower and broccoli bake; cheese, onion and potato pie) and four meat options (chilli con carne; beef burger; roast turkey; beef and mushroom pie). Meat and meat-free options were matched by meal type (e.g. chilli), and as closely as possible for accompanying sides (see supplementary materials for images used). Pictures of main meals were taken from a manual used by worksite cafeterias for a major supermarket chain. These pictures showed meals as made in these cafeterias, with their ingredients provided in the manual.

##### Socioeconomic status

The primary measure was highest educational qualification, subdivided into two groups: higher (2+ A-Levels or equivalent, or above) vs. lower (up to GCSE-level education/1 A-level or equivalent). A-levels are typically taken at around age 18 in the UK, and represent qualifications that would be recognised as entry requirements to higher education, while GCSEs (General Certificate of Secondary Education) are a less advanced qualification, usually taken at around age 16. Annual household income and postcode (to determine their local Index of Multiple Deprivation) were also collected. Education was selected as the primary measure, given education may be indicative of skills and knowledge to avoid harmful behaviours [12, 13], while income and area-level deprivation may be less likely to impact on behaviour in experiments where no payment is made and which are conducted online.

#### Procedure

Upon accessing the online survey, participants provided consent, and then completed measures relating to basic demographics used in quotas (age, gender and highest educational qualification).

**Fig. 1:**
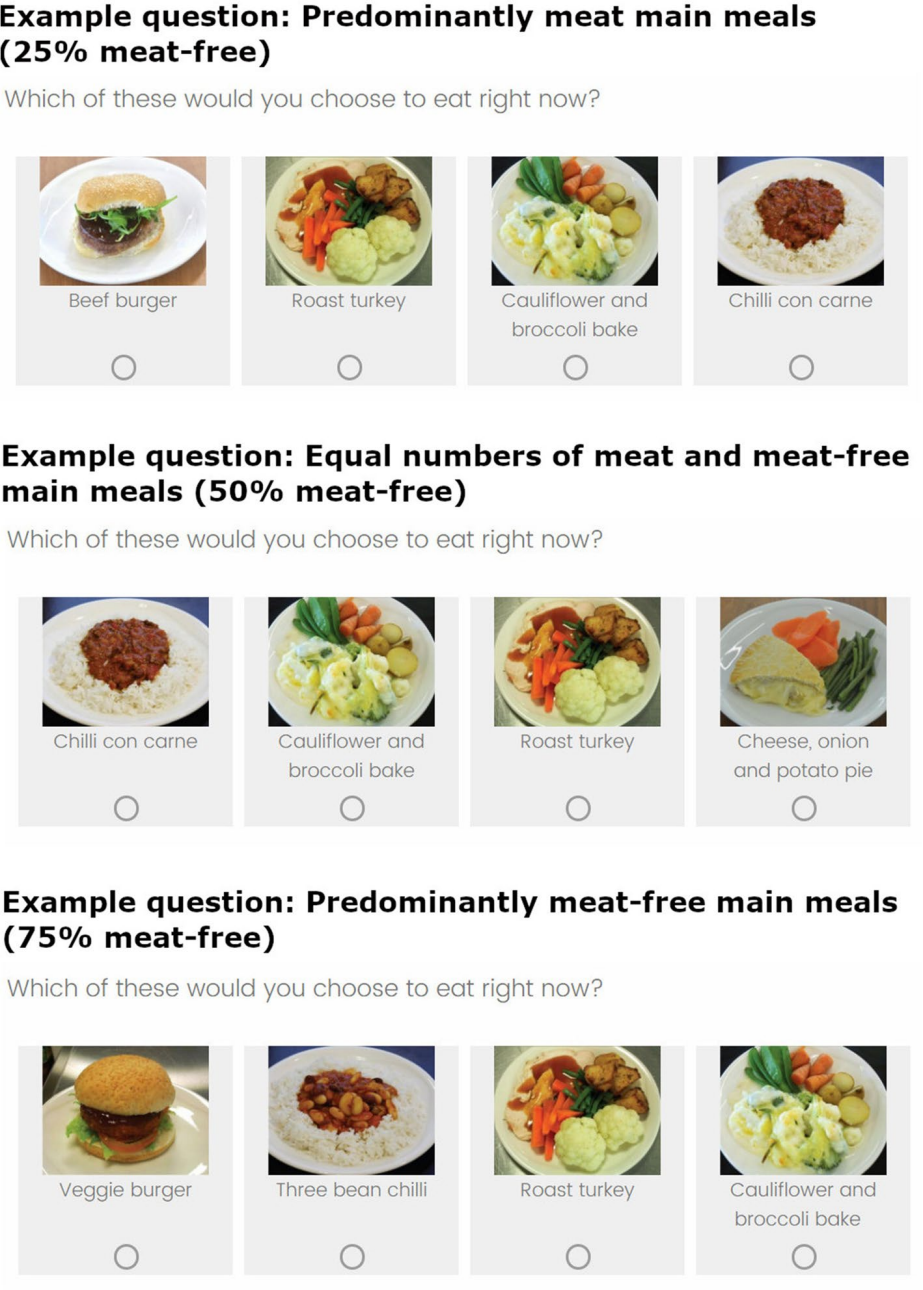
Example questions from each of the three availability conditions

Each participant was then randomised (via the randomisation procedures on the Qualtrics survey platform) to see a set of four meal options, corresponding to one of the three availability conditions: (1) “predominantly meat”; (2) “equal numbers”; (3) “predominantly meat-free” (see Fig. 1). The food options that appeared in the presented set were randomised (i.e. these could be any combination of the four meat and four meat-free options used in the study). Food options were also randomised to their position in the display (far right, middle right, middle left, far left). Participants were asked to select the option they would prefer to eat right now from the presented set of options.

Finally, participants completed measures on household income, postcode, hunger and baseline consumption of meat-based main meals.

#### Analyses

##### Impact of the intervention

Logistic regression analyses compared whether (a) predominantly-meat options, (b) equal numbers of options or (c) predominantly meat-free options alter the likelihood of participants’ selecting meat-free options. Covariates included age, gender, education, hunger, baseline consumption of meat-based main meals.

##### Differential impact of the intervention

The primary analysis was repeated, including interactions between: (1) availability condition and baseline consumption of meat-based main meals; (2) availability condition and highest educational qualification; (3) availability condition and gender.

##### Secondary analyses

These analyses explored using alternative SES indicators (Household income group: (1) Up to £17,499 per year; (2) £17,500-£29,999 per year; (3) £30,000-£49,999 per year; (4) £50,000 or more per year; Index of Multiple Deprivation: quintiles) instead of education in the above analysis for Aim 2.

## Results

### Study 1: natural experiment in a university cafeteria

#### Impact on sales of meat-based meals

**Fig. 2:**
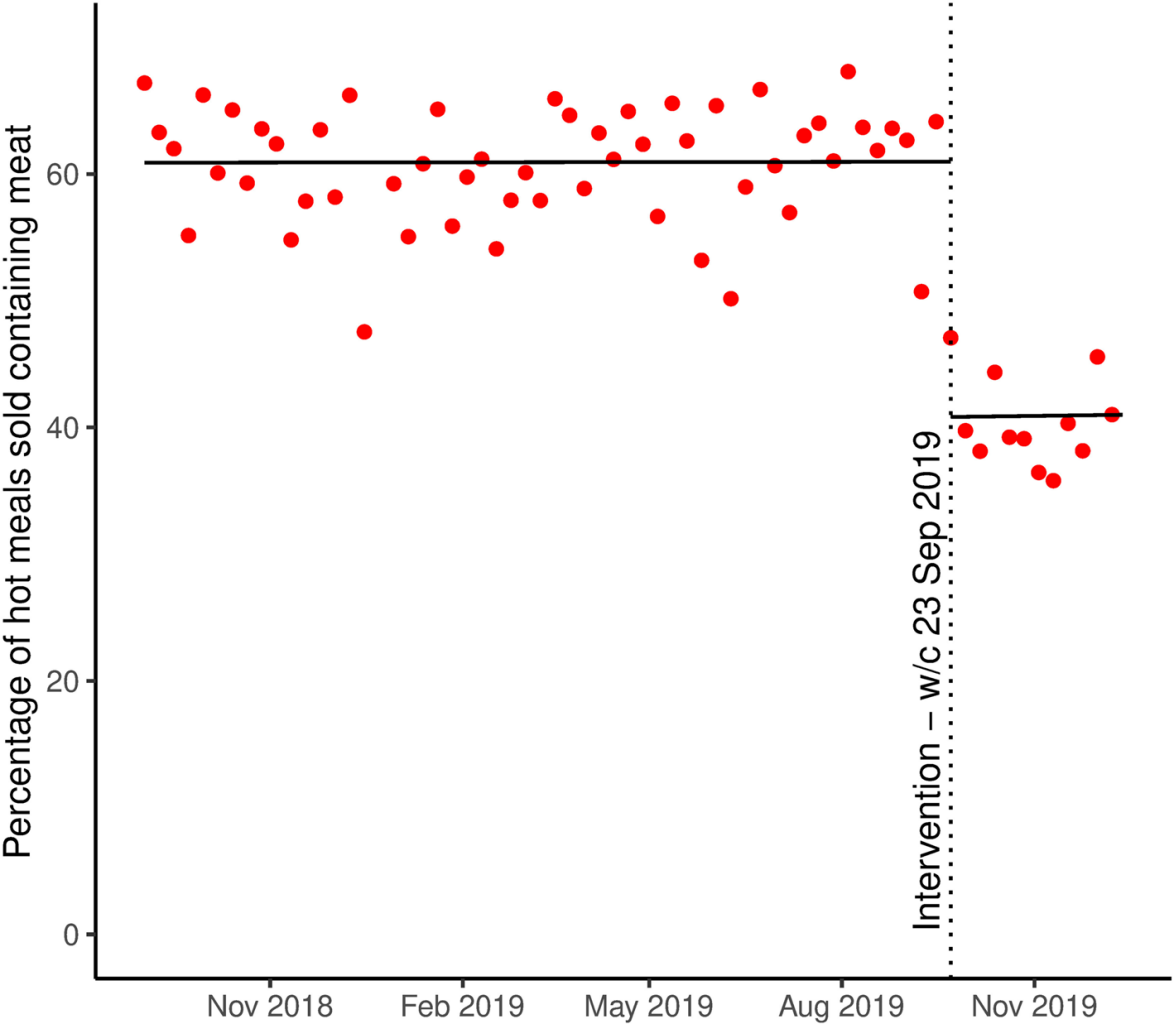
Percentage of hot meals containing meat sold in the intervention site, by week. Points represent observations and solid lines indicate model predictions

There was a 19.9 percentage point decrease (95%CIs:-25.2, − 14.6) in the proportion of sales comprised by meat options in the intervention cafeteria when a greater proportion of available meals were meat-free (see Fig. 2; Supplementary Table S1 for full model). There was no evidence from any of the control cafeterias of a difference in sales between the baseline and intervention periods (see Table 1).

**Table 1:** Percentage of hot meals containing meat sold per week: baseline and intervention means, and model results testing the effect of the availability intervention, by cafeteria site

	Mean percentage of hot meals sold per week (s.d.)		Model results^a^	
	Baseline	Intervention	Intervention coefficient (95% CIs)	*P* value of intervention effect
Intervention site	60.74 (4.57)	39.19 (2.94)	− 19.88 (− 25.17, − 14.60)	< 0.001
Control site 1	57.29 (8.12)	50.04 (5.79)	0.04 (− 5.84, 5.92)	0.672
Control site 2	43.55 (13.16)	56.29 (5.97)	7.35 (− 2.19, 16.90)	0.222
Control site 3	54.97 (6.08)	53.08 (7.41)	−0.21 (−7.92, 7.50)	0.720
Control site 4	52.57 (4.55)	49.07 (7.13)	−2.79 (− 10.27, 4.69)	0.752
Control site 5	50.97 (5.39)	49.07 (6.17)	1.04 (−6.68, 8.76)	0.162
Control site 6	52.23 (8.53)	44.38 (6.06)	−2.45 (−11.13, 6.23)	0.381
Control site 7	57.10 (5.51)	56.89 (7.74)	−2.56 (− 8.40, 3.28)	0.353
Control site 8	49.85 (4.44)	47.81 (5.91)	−1.94 (−9.63, 5.76)	0.372
Control site 9	48.38 (9.68)	44.55 (7.61)	−2.37 (−11.64, 6.91)	0.480
Control site 10	49.61 (5.17)	47.04 (9.14)	−1.60 (− 10.62, 7.42)	0.490
Control site 11	51.59 (10.00)	45.22 (7.45)	−6.03 (−12.35, 0.29)	0.074

^a^ Interrupted time series predicting the percentage of hot meals containing meat purchased per week, depending on the relative availability of meat-free meals (modelled using a dummy variable). A dummy variable indicating whether it was during university term (vs. holidays) was included as a covariate

##### Daily analysis

Similar results were obtained in the sensitivity analysis using daily data (− 19.10; 95% CIs: − 28.00, − 10.21; *p*-value < 0.001; Supplementary Table S1).

#### Impact on sales of all lunchtime options containing meat

The percentage of total meals containing meat sold per week in the intervention cafeteria fell from a mean of 58.2 (s.d. 3.42) at baseline to 38.7 (s.d. 3.29) during the intervention period. Interrupted time series analyses suggested the percentage of meals purchased containing meat decreased by 20 percentage points (− 20.3; 95%CIs: − 25.1, − 15.5; *p* < 0.001; Supplementary Table S1) when the availability of meat-free meals increased.

#### Impact on total sales

The mean total number of meals sold per week was 1183.7 (s.d. 371.0) during baseline and 1580.2 (s.d. 335.6) during the intervention period. Analyses suggested that the total number of meals sold increased during the intervention period (by 544.4 meals; 95%CIs: 200.6, 888.2; *p* < 0.001; Supplementary Table S1).

### Study 2: natural experiment in worksite cafeterias

#### Multilevel regression analyses

The mean percentage of meat-free meals purchased in the 8 weeks prior to the menu change was 9.6% (s.d. 8.4), rising to 12.4% (s.d. 10.8) in the 8 weeks after the change. Multilevel regressions found no conclusive evidence of a difference in purchasing of meat-free meals, with a 2.3 percentage point increase (95%CIs: − 1.3, 5.9; *p* = 0.204) after the menu change.

The mean percentage of meat-free meal options offered in the 8 weeks prior to the menu change was 21.0% (s.d. 12.7), rising to 23.8% (s.d. 12.4) in the 8 weeks after. Multilevel regressions suggested an increase of 4.3 percentage points following the menu change (95%CIs: 0.6, 8.0; *p* = 0.025).

##### Sensitivity analysis

There was considerable variability in implementation across sites (see Supplementary Figs. S2 and S3). In 7 of the 18 sites the mean percentage of meat-free meals offered decreased after the menu change (mean difference [before minus after] = − 2.8; s.d. 5.6; range − 16.1, 5.2). A sensitivity analysis examined the impact of the menu change within the 10 sites for which at least a one percentage point increase was observed in the percentage of meat-free meals offered after the menu change. This multilevel regression suggested that the percentage of meat-free meals purchased in these sites increased by 5.4 percentage points (95%CIs: 0.2, 10.5; *p* = 0.043) after the menu change. The mean difference in percentage of meat-free meals offered in these 10 sites was − 6.7 (s.d. 4.4; range − 16.1, − 1.6). Supplementary Table S4 gives the full coefficients from all the multilevel regression models.

#### Interrupted time-series analyses

**Fig. 3:**
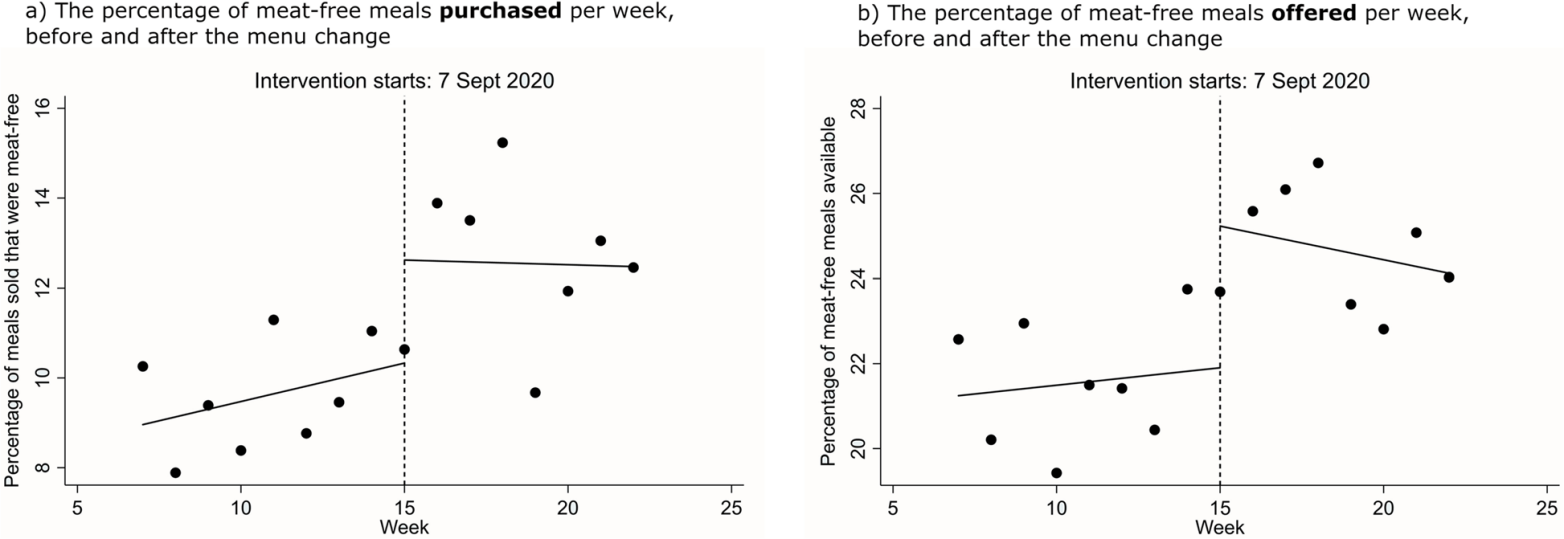
Interrupted time-series models of: **a** The percentage of meat-free meals purchased per week, before and after the menu change. **b** The percentage of meat-free meals offered per week, before and after the menu change. Points represent observations and solid lines indicate model predictions

Interrupted time-series models, using data aggregated across the 18 sites, and including observations where zero meat-free meals were offered, were also conducted. These analyses suggested that the percentage of meat-free meals purchased increased post-menu change (2.3 percentage points, 95%CIs: 0.4, 4.2, *p* = 0.020). There was no evidence of either a time trend (0.17, 95%CIs: − 0.02, 0.37, *p* = 0.079) or a change in time trend following the menu change (− 0.19, 95%CIs: − 0.55, 0.17, *p* = 0.268) (see Fig. 3a).

For the secondary outcome, interrupted time-series models again suggested the percentage of meat-free meals offered increased after the menu change (3.3 percentage points, 95%CIs: 0.9, 5.8, *p* = 0.012). There was no evidence suggesting a time trend (0.08, 95%CIs: − 0.28, 0.44, *p* = 0.630) or a change in time trend after the menu change (− 0.24, 95%CIs: − 0.64, 0.15, *p* = 0.209) (see Fig. 3b).

##### Sensitivity analysis

An interrupted time-series model, conducted on the 10 sites that showed at least a one percentage point increase in the percentage of meat-free meals offered, showed that the percentage of meat-free meals purchased increased after the menu change by 5.3 percentage points (95%CIs: 2.0, 8.6, *p* = 0.004). This analysis suggested a decreasing trend over time (− 0.32, 95%CIs: − 0.57, − 0.07, *p* = 0.017), but no evidence of a change in time trend following the menu change (0.54, 95%CIs: − 0.04, 1.12, *p* = 0.066) (see Supplementary Fig. S5).

### Study 3: online experiment

Of the 2205 participants completing the survey, 4 completed the study in less than 30% of the median time, and were excluded as speeders (see Supplementary Materials for CONSORT Flow Diagram). The mean age of the final sample of 2201 participants was 46.9 (range 18-98), with an even split between females and males and higher vs. lower education (see Supplementary Table S6 for participant characteristics by study condition).

#### Impact of availability condition

When shown equal numbers of meat and meat-free options, 28.5% of participants selected a meat-free option, falling to 12.4% in the predominantly meat condition, and rising to 47.5% in the predominantly meat-free condition.

Logistic regression analyses (Table 2) showed that reducing meat-free options from 50 to 25% reduces participants’ selection of meat-free options, with the odds of selecting a meat-free option decreasing by a factor of 0.35 (95% CIs: 0.26, 0.46). Conversely, increasing meat-free options from 50 to 75% more than doubles the odds of selecting a meat-free option, with an odds ratio (OR) of 2.43 (95% CIs: 1.94, 3.04).

**Table 2:** Coefficients from logistic regression predicting selection of a meat-free option^a^

		Odds Ratio	95% Confidence Intervals		*p*
Meat-free Availability *[Reference Group: 50% meat free]*	25% meat free	0.35	0.26	0.46	< 0.001
	75% meat free	2.43	1.94	3.04	< 0.001
Age		1.009	1.003	1.016	0.005
Gender *[Reference Group: Male]*	Female	1.61	1.31	2.00	< 0.001
	Other	3.44	0.63	18.87	0.154
Education *[Reference Group: Higher education]*	Lower education	0.65	0.53	0.79	< 0.001
Hunger ^b^		0.999	0.928	1.075	0.97
Usual meat consumption score ^c^		0.79	0.75	0.83	< 0.001
Constant		0.96	0.59	1.57	0.87

^a^ Number of observations = 2199; Pseudo R-squared = 0.1291

^b^Hunger was measured on a scale from −3 (“Very full”) to 3 (“Very hungry”)

^c^Usual meat consumption score (taking values between 0 and 10) was calculated by summing participants’ self-reported usual meat consumption at lunchtimes and dinnertimes (for each question, scores of 0 are assigned to answers of “Never”, 1 for “Less than once a week”, 2 “1-2 times a week”, 3 “3-4 times a week”, 4 “5-6 times a week” and 5 “Every day”). Two participants had missing values for this score

Older (OR: 1.009; 95%CIs: 1.003, 1.016) and female (OR: 1.61; 95% CIs: 1.31, 2.00) participants had higher odds of selecting a meat-free option, while lower education (OR: 0.65; 95% CIs: 0.53, 0.79) and higher levels of usual meat consumption (OR: 0.79; 95% CIs: 0.75, 0.83) were associated with lower odds of selecting a meat-free option.

No evidence was found to suggest an interaction between meat-free availability and any of the investigated participant characteristics (usual meat consumption; education; gender; Supplementary Table S7) or alternative socioeconomic status variables (income and Index of Multiple Deprivation; Supplementary Tables S8 and S9). For these alternative indicators there was no consistent patterning with the odds of selecting a meat-free option (Supplementary Figs. S10-S12).

## Discussion

These three studies provide support to the hypothesis that altering the relative availability of meat vs. meat-free options can be effective at reducing selection or purchasing of meat if sufficient changes are made to availability. The effect size is uncertain due to differences in the degree to which availability was altered, but there was no evidence from a nationally representative survey that the effectiveness of the intervention varied by gender, education, income, area-level deprivation or meat consumption habits.

Strengths of this research include the combination of natural experiments of real-world purchasing, and a tightly-controlled experimental study using a large UK-representative sample, allowing evaluation across demographic groups. Studies 1 and 2 provide further evidence of the impact of altering meat-free availability in real-world purchasing contexts. The inclusion of the control cafeterias in Study 1 strengthens these findings, by demonstrating that they are unlikely to have occurred without intervention and in Study 2 we showed some, albeit smaller, impact in worksites catering to a broader demographic than has previously been investigated. Moreover, Study 3 allowed investigation of the impact of availability across a range of participant demographics. Investigations of potential compensatory behaviour also provide insight into the implementation impact, with no evidence of increased meat-based sales from other lunch options, nor any decline in total sales, suggesting no decrease in footfall in Study 1 (due to changing circumstances resulting from the Covid-19 pandemic during the study period, equivalent analyses were not conducted for Study 2).

However, since Studies 1 and 2 were natural experiments, it is not possible to rule out certain potential biases that may have influenced these findings (e.g. in Study 1 the cafeteria was selected to trial this change, perhaps due to the likely receptiveness of its customers). Study 2 was conducted during the Covid-19 pandemic, leading to possible fluctuations in employees at the sites, although sites were selected on the basis that they were open throughout this period. Moreover, given that the analyses for Study 2 relied on purchase data, items that were offered but never purchased were not included in this dataset, which could have truncated the data. Key limitations of Study 3 were the hypothetical food selection – participants did not receive their selected meal – and the limited range of main meal options used in the task. The key limitations of these studies are somewhat offset by the finding of similar results across the set of studies, which counteract some of each others’ weaknesses.

In Study 1 levels of meat-free purchasing broadly mapped onto the proportion of meat-free meals available (when 33% of options were meat-free, approximately 40% of purchased meals were meat-free, and when 67% of options were meat-free, around 60% of purchased meals were meat-free). However, in the worksite cafeterias in Study 2 and the representative sample in Study 3, participants tended to select meat options more than would be expected purely based on availability. In Study 2, between 10 and 12% of sales were meat-free, with 21-24% availability – and likewise in Study 3 where 12% of participants selected a meat-free option in the predominantly meat condition (25% meat-free), compared to 29% in the equal numbers condition (50% meat-free), and 48% in the predominantly meat-free condition (75% meat-free). This is similar to the previous experimental study in a university cafeteria, where decreasing the proportion of meat-based meals from 75 to 50% decreased their sales from 81 to 73% [9]. This strong tendency to purchase meat-based meals, well in excess of the proportion available, highlights the social and cultural norms which favour meat consumption [14, 15].

The effect size in Study 2 was much lower than suggested in the Study 1 analysis or in the online experiment. However there was poor implementation of the intervention, with only half the sites taking on the new meat-free menu options, and so little change in availability. Our sensitivity analyses suggested that meat-free meal sales did increase within those sites where availability increased following the menu change, though the magnitude of the change was still considerably smaller than Study 1. In addition, the customers using the cafeteria in Study 1 were likely to be highly educated and may have had lower than average levels of meat consumption, but the sites in Study 2 were catering to workers in distribution and manufacturing centres and had higher meat purchasing in general. We hypothesised that this may contribute to the difference in effect. However, the online experiment found no evidence of any differential responsiveness to the availability intervention by gender, socioeconomic status or usual meat consumption. This suggests targeting the relative availability of meat-free options has the potential for broad effectiveness, without creating or exacerbating inequalities. This could tie in with suggestions that micro-environmental interventions – such as altering the availability of healthier vs. less-healthy options – may rely largely on automatic processes, and may be more likely to be equitable as a result [16, 17]. This study contributes to the as yet limited evidence for this hypothesis, with mixed evidence from two recent systematic reviews of the equity of dietary nudges [18, 19]. Further exploration of potential moderators of the impact of availability such as preferences for the meals involved or social norms within sites could help to understand possible differences in effectiveness between sites or if there are contexts where socioeconomic differences may occur [20].

## Conclusion

These preliminary studies show that changing the availability of meat-free options may be a promising intervention to reduce the selection and purchase of meals containing meat, with no evidence of differential effectiveness across population subgroups when directly tested in an online study. While natural experiments provide an efficient research opportunity, field studies with more robust designs are needed to test the effectiveness of increasing availability of meat-free meals. Further qualitative research is also required exploring the acceptability of, or potential barriers to, chefs implementing a shift towards greater availability of meat-free meals when baseline meat consumption is high.

## Supplementary Information

**Additional file 1.**

## Data Availability

Data from the natural experiments are not available for sharing due to commercial sensitivities. The dataset from Study 3 is available in the Open Science Framework [https://osf.io/24cb9/].
