# Supplementary material for: Impact of increasing the relative availability of meat-free options on food selection: two natural field experiments and an online randomised trial
Source: Int J Behav Nutr Phys Act. 2022 Jan 31;19:9. doi: 10.1186/s12966-021-01239-z (PMC8801279; doi:10.1186/s12966-021-01239-z)
Supplement: Supplementary file 1 — Additional file 1. [file 12966_2021_1239_MOESM1_ESM.docx]

**Supplementary Materials:**

***Impact of increasing the relative availability of meat-free options on food selection: Two natural field experiments and an experimental online study***

**Study 1**

**Supplementary Table S1.** Full model results testing the effect of the availability intervention on primary and secondary outcomes in the intervention cafeteria.

|  | Percentage of hot meals containing meat | | | | Percentage of all lunchtime options containing meat | | Number of total lunchtime options sold | |
| --- | --- | --- | --- | --- | --- | --- | --- | --- |
|  | Per week | | Per day | | Per week | | Per week | |
|  | Coefficient  (95% CIs) | p value | Coefficient  (95% CIs) | p value | Coefficient  (95% CIs) | p value | Coefficient  (95% CIs) | p value |
| Primary Outcome | | | | | | | | |
| Availability intervention | **-19.88** (-25.17, -14.60) | <0.001 | - | - | - | - | - | - |
| Term | -2.11 (-4.37, 0.15) | 0.067 | - | - | - | - | - | - |
| Secondary Outcomes | | | | | | | | |
| Availability intervention | **-19.76** (-25.00, -14.51) | <0.001 | **-19.10** (-28.00, -10.21) | <0.001 | **-20.30** (-25.10, -15.49) | <0.001 | **544.43** (200.63, 888.24) | 0.002 |
| Term | - | - | -1.97 (-4.82, 0.88) | 0.176 | - | - | **343.38** (187.08, 499.68) | <0.001 |
| Day of the week | - | - | 0.87 (-0.34, 2.09) | 0.159 | - | - | - | - |

*Bold font indicates model coefficients significant at p<0.05*

**Study 2**

**Supplementary Figure S2**. Percentage of vegetarian meals purchased per week in each of the 18 sites (red line indicates menu change)

**Supplementary Figure S3**. Percentage of vegetarian meals offered each week in each of the 18 sites (red line indicates menu change)

**Supplementary Table S4**. Results from multilevel regression models of the impact of the menu change on the percentage of meals purchased that were vegetarian (95% CIs)

|  | **Primary analysis** | | **Sensitivity analysis** |
| --- | --- | --- | --- |
|  | **Percentage vegetarian purchased per week** | **Percentage vegetarian offered per week** | **Percentage vegetarian purchased per week** |
| Menu change | 2.32 (-1.26, 5.90) | **4.25** (0.55, 7.96) | **5.35** (0.16, 10.54) |
| Mean % vegetarian sales at baseline | **0.67** (0.39, 0.95) | **1.04** (0.68, 1.40) | **0.57** (0.15, 0.99) |
| Week | 0.08 (-0.31, 0.47) | -0.14 (-0.55, 0.26) | -0.14 (-0.70, 0.43) |
| Constant | 2.42 (-2.96, 7.80) | 12.9 (6.72, 19.0) | 5.59 (-2.75, 13.9) |
| *Random effects* |  |  |  |
| Site (constant) | 13.3 (5.8, 30.4) | 24. 1 (11.3, 51.7) | 19.8 (6.8, 57.6) |
| Residual | 56.6 (47.6, 67.3) | 60.8 (51.1, 72.3) | 67.6 (53.7, 85.1) |
| *n* | 272 observations  (18 sites, between 11-16 weeks/site) | | 155 observations (10 sites, between 14-16 weeks/site) |

*Bold font indicates model coefficients significant at p<0.05*

**Supplementary Figure S5**. Interrupted time-series model of the percentage of vegetarian meals purchased per week, for the 10 sites with at least a 1-percentage point increase in the percentage of vegetarian meals available per week

**Study 3**

**Supplementary Table S6.** Participant characteristics by study condition (% (n), unless otherwise stated)

|  | | Availability condition | | | Total |
| --- | --- | --- | --- | --- | --- |
|  |  | Predominantly meat | Equal numbers | Predominantly meat-free |  |
| n | | 735 | 737 | 729 | 2201 |
| Age (Mean (s.d.)) | | 47.1 (17.1) | 46.6 (16.6) | 46.9 (16.7) | 46.9 (16.8) |
| Gender | Male | 51.7 (380) | 47.1 (347) | 50.6 (369) | 49.8 (1096) |
|  | Female | 47.9 (352) | 52.6 (388) | 49.1 (358) | 49.9 (1098) |
|  | Other | 0.4 (3) | 0.3 (2) | 0.3 (2) | 0.3 (7) |
| Ethnicity | White | 90.6 (666) | 89.4 (659) | 92.3 (673) | 90.8 (1998) |
|  | Other | 8.6 (63) | 9.5 (70) | 6.7 (49) | 8.3 (182) |
|  | Prefer not to say | 0.8 (6) | 1.1 (8) | 1.0 (7) | 1.0 (21) |
| Highest educational qualification | Lower (up to 1 A-level or equivalent) | 50.3 (370) | 49.0 (361) | 50.8 (370) | 50.0 (1101) |
|  | Higher (2+ A-Levels or equivalent, or above) | 49.7 (365) | 51.0 (376) | 49.2 (359) | 50.0 (1100) |
| Annual household income | Up to £17,499 | 26.3 (193) | 25.5 (188) | 28.4 (207) | 26.7 (588) |
|  | £17,500-£29,999 | 23.4 (172) | 23.5 (173) | 21.8 (159) | 22.9 (504) |
|  | £30,000-£49,999 | 26.1 (192) | 24.3 (179) | 26.3 (192) | 25.6 (563) |
|  | £50,000 or more | 20.0 (147) | 21.8 (161) | 18.7 (136) | 20.2 (444) |
|  | Don’t know/ prefer not to say | 4.2 (31) | 4.9 (36) | 4.8 (35) | 4.6 (102) |
| Index of Multiple Deprivation^1^ | Quintile 1 | 16.9 (124) | 15.7 (116) | 13.2 (96) | 15.3 (336) |
|  | Quintile 2 | 14.1 (104) | 15.1 (111) | 16.5 (120) | 15.2 (335) |
|  | Quintile 3 | 13.5 (99) | 15.3 (113) | 16.9 (123) | 15.2 (335) |
|  | Quintile 4 | 15.0 (110) | 16.0 (118) | 14.7 (107) | 15.2 (335) |
|  | Quintile 5 | 15.9 (117) | 15.1 (111) | 14.7 (107) | 15.2 (335) |
|  | Missing | 24.6 (181) | 22.8 (168) | 24.1 (176) | 23.9 (525) |
| Hunger (Mean (s.d.)) ^2^ | | 0.4 (1.5) | 0.5 (1.4) | 0.5 (1.3) | 0.5 (1.4) |
| Usual meat consumption ^3^ (Mean (s.d.)) | | 6.0 (2.0) | 6.1 (2.0) | 6.0 (2.0) | 6.0 (2.0) |

*^1^ Index of Multiple Deprivation is a measure of relative area-level deprivation; Quintile 1 represents the most deprived areas and Quintile 5 the least deprived (see:* [*https://assets.publishing.service.gov.uk/government/uploads/system/uploads/attachment_data/file/464430/English_Index_of_Multiple_Deprivation_2015_-_Guidance.pdf*](https://assets.publishing.service.gov.uk/government/uploads/system/uploads/attachment_data/file/464430/English_Index_of_Multiple_Deprivation_2015_-_Guidance.pdf)*)*

*^2^ Hunger was measured on a scale from -3 (“Very full”) to 3 (“Very hungry”).*

*^3^ Usual meat consumption score (taking values between 0-10) was calculated by summing participants’ self-reported usual meat consumption at lunchtimes and dinnertimes (for each question, scores of 0 are assigned to answers of “Never”, 1 for “Less than once a week”, 2 “1-2 times a week”, 3 “3-4 times a week”, 4 “5-6 times a week” and 5 “Every day”). Meat consumption scores were missing for two participants (one in the equal numbers condition and one in the predominantly meat-free condition)*

**Supplementary Table S7.** Coefficients from logistic regressions predicting selection of a meat-free option with interaction effects

|  | | | Interactions by gender | | | Interactions by education | | | Interactions by usual meat consumption | | |
| --- | --- | --- | --- | --- | --- | --- | --- | --- | --- | --- | --- |
|  | | | Odds Ratio | 95% Confidence  Intervals | | Odds Ratio | 95% Confidence  Intervals | | Odds Ratio | 95% Confidence  Intervals | |
| Meat-free Availability *[Ref: 50% meat free]* | | 25% meat free | **0.39** | 0.26 | 0.60 | **0.39** | 0.27 | 0.57 | **0.37** | 0.16 | 0.87 |
|  |  | 75% meat free | **2.67** | 1.91 | 3.73 | **2.64** | 1.93 | 3.62 | 1.61 | 0.78 | 3.32 |
| Age | | | **1.01** | 1.00 | 1.02 | **1.01** | 1.00 | 1.02 | **1.01** | 1.00 | 1.02 |
| Gender *[Reference Group: Male]* | | Female | **1.84** | 1.31 | 2.59 | **1.62** | 1.31 | 2.00 | **1.62** | 1.31 | 2.00 |
|  |  | Other | - | - | - | 3.54 | 0.64 | 19.58 | 3.50 | 0.64 | 19.27 |
| Gender * Availability | | Female * 25% meat free | 0.79 | 0.45 | 1.38 | - | - | - | - | - | - |
|  |  | Female * 75% meat free | 0.83 | 0.53 | 1.30 | - | - | - | - | - | - |
| Education *[Ref: Higher education]* | | Lower education | **0.65** | 0.53 | 0.80 | **0.74** | 0.53 | 1.03 | **0.65** | 0.53 | 0.79 |
| Education * Availability | | Lower education* 25% meat free | - | - | - | 0.77 | 0.44 | 1.35 | - | - | - |
|  |  | Lower education* 75% meat free | - | - | - | 0.84 | 0.54 | 1.31 | - | - | - |
| Hunger | | | 1.00 | 0.93 | 1.08 | 1.00 | 0.93 | 1.08 | 1.00 | 0.93 | 1.08 |
| Usual meat consumption score (UMCS) | | | **0.79** | 0.75 | 0.84 | **0.79** | 0.75 | 0.83 | **0.77** | 0.70 | 0.84 |
| Usual meat consumption score*Availability | UMCS* 25% meat free | | - | - | - | - | - | - | 0.99 | 0.85 | 1.14 |
|  | UMCS* 75% meat free | | - | - | - | - | - | - | 1.07 | 0.94 | 1.21 |
| Constant | | | 0.88 | 0.52 | 1.47 | 0.90 | 0.54 | 1.50 | 1.12 | 0.59 | 2.12 |
| *Number of observations; Pseudo R-squared* | | | 2192; 0.13 | | | 2199; 0.13 | | | 2199; 0.13 | | |

*Bold font indicates model coefficients significant at p<0.05*

**Supplementary Table S8.** Coefficients from logistic regressions predicting selection of a meat-free option with income rather than education

|  | | Income | | | Interactions by income | | |
| --- | --- | --- | --- | --- | --- | --- | --- |
|  | | Odds Ratio | 95% Confidence  Intervals | | Odds Ratio | 95% Confidence  Intervals | |
| Meat-free Availability *[Reference Group: 50% meat free]* | 25% meat free | **0.34** | 0.25 | 0.45 | **0.30** | 0.18 | 0.50 |
|  | 75% meat free | **2.43** | 1.93 | 3.06 | **1.96** | 1.28 | 2.99 |
| Age | | **1.01** | 1.00 | 1.01 | **1.01** | 1.00 | 1.01 |
| Gender *[Reference Group: Male]* | Female | **1.56** | 1.26 | 1.94 | **1.57** | 1.27 | 1.95 |
|  | Other | 4.72 | 0.73 | 30.59 | 4.72 | 0.73 | 30.68 |
| Income *[Reference Group: Up to £17,499]* | £17,500-£29,999 | 0.91 | 0.68 | 1.21 | 0.85 | 0.53 | 1.35 |
|  | £30,000-£49,999 | 1.04 | 0.79 | 1.37 | 0.75 | 0.47 | 1.21 |
|  | £50,000+ | 1.20 | 0.90 | 1.61 | 1.08 | 0.68 | 1.72 |
| Income * Availability | £17,500-£29,999 *  25% meat free | - | - | - | 1.05 | 0.47 | 2.33 |
|  | £30,000-£49,999 *  25% meat free | - | - | - | 1.57 | 0.73 | 3.35 |
|  | £50,000+ *  25% meat free | - | - | - | 0.96 | 0.42 | 2.17 |
|  | £17,500-£29,999 *  75% meat free | - | - | - | 1.13 | 0.60 | 2.14 |
|  | £30,000-£49,999 *  75% meat free | - | - | - | 1.66 | 0.89 | 3.10 |
|  | £50,000+ *  75% meat free | - | - | - | 1.31 | 0.68 | 2.50 |
| Hunger | | 1.00 | 0.93 | 1.08 | 1.01 | 0.94 | 1.09 |
| Usual meat consumption score | | **0.79** | 0.75 | 0.84 | **0.79** | 0.75 | 0.84 |
| Constant | | 0.85 | 0.50 | 1.44 | 0.96 | 0.54 | 1.70 |
| *Number of observations; Pseudo R-squared* | | 2097; 0.13 | | | 2097; 0.13 | | |

*Bold font indicates model coefficients significant at p<0.05*

**Supplementary Table S9.** Coefficients from logistic regressions predicting selection of a meat-free option with Index of Multiple Deprivation

|  | | Index of Multiple Deprivation (IMD) | | | Interactions by IMD | | |
| --- | --- | --- | --- | --- | --- | --- | --- |
|  | | Odds Ratio | 95% Confidence  Intervals | | Odds Ratio | 95% Confidence  Intervals | |
| Meat-free Availability *[Reference Group: 50% meat free]* | 25% meat free | **0.36** | 0.26 | 0.49 | **0.39** | 0.20 | 0.77 |
|  | 75% meat free | **2.37** | 1.84 | 3.07 | **2.67** | 1.48 | 4.84 |
| Age | | **1.01** | 1.00 | 1.01 | **1.01** | 1.00 | 1.01 |
| Gender *[Reference Group: Male]* | Female | **1.57** | 1.24 | 2.00 | **1.58** | 1.24 | 2.01 |
|  | Other | 5.36 | 0.11 | 266.79 | 5.55 | 0.10 | 312.71 |
| Index of Multiple Deprivation *[Reference Group: Quintile 1 (most deprived)]* | Quintile 2 | 0.97 | 0.68 | 1.40 | 1.01 | 0.57 | 1.88 |
|  | Quintile 3 | 0.94 | 0.66 | 1.35 | 1.27 | 0.71 | 2.29 |
|  | Quintile 4 | 0.95 | 0.66 | 1.37 | 1.06 | 0.59 | 1.92 |
|  | Quintile 5 | 0.97 | 0.67 | 1.40 | 0.81 | 0.44 | 1.49 |
| Index of Multiple Deprivation * Availability | Quintile 2 * 25% meat free | - | - | - | 1.14 | 0.43 | 3.01 |
|  | Quintile 3 * 25% meat free | - | - | - | 0.39 | 0.13 | 1.17 |
|  | Quintile 4 * 25% meat free | - | - | - | 0.79 | 0.29 | 2.14 |
|  | Quintile 5 * 25% meat free | - | - | - | 1.55 | 0.60 | 4.00 |
|  | Quintile 2 * 75% meat free | - | - | - | 0.82 | 0.36 | 1.86 |
|  | Quintile 3 * 75% meat free | - | - | - | 0.70 | 0.31 | 1.56 |
|  | Quintile 4 * 75% meat free | - | - | - | 0.84 | 0.37 | 1.92 |
|  | Quintile 5 * 75% meat free | - | - | - | 1.19 | 0.51 | 2.75 |
| Hunger | | 0.97 | 0.89 | 1.05 | 0.97 | 0.89 | 1.05 |
| Usual meat consumption score | | **0.79** | 0.74 | 0.84 | **0.79** | 0.74 | 0.84 |
| Constant | | 0.95 | 0.50 | 1.80 | 0.89 | 0.44 | 1.80 |
| *Number of observations; Pseudo R-squared* | | 1674; 0.12 | | | 1674; 0.13 | | |

*Bold font indicates model coefficients significant at p<0.05*

**Supplementary Figure S10**. Predicted probability of selecting a meat-free option by meat-free availability and usual meat consumption

**Supplementary Figure S11**. Predicted probability of selecting a meat-free option by meat-free availability and education

**Supplementary Figure S12**. Predicted probability of selecting a meat-free option by meat-free availability and gender

**Supplementary File S13.** Images used in the online study

| **Meat** | **Meat-free** |
| --- | --- |
| Chilli con carne   | Three bean chilli   |
| Beef Burger  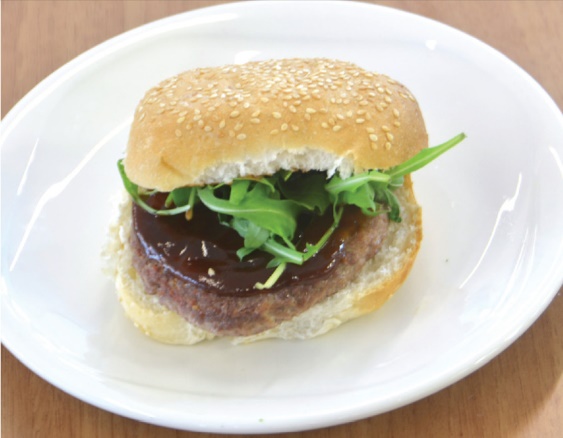 | Veggie Burger  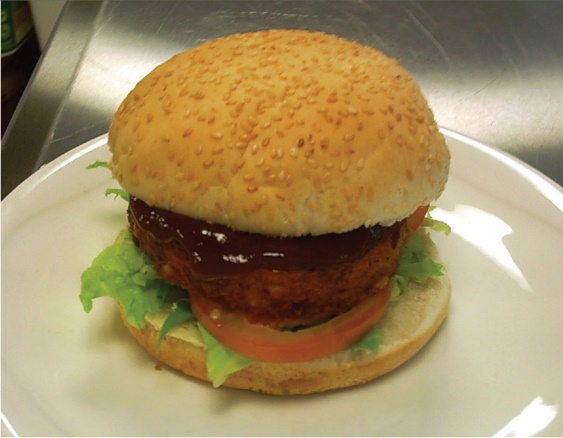 |
| Roast Turkey  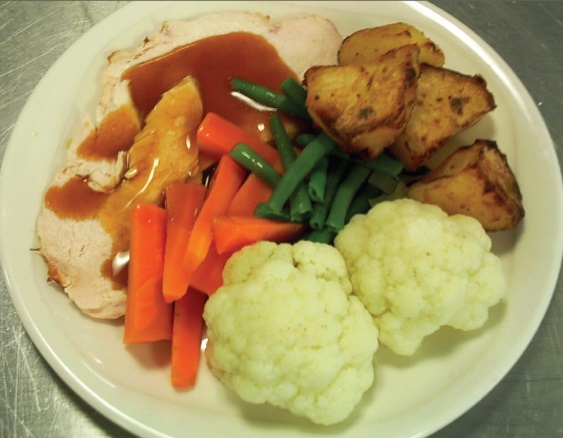 | Cauliflower and Broccoli Bake  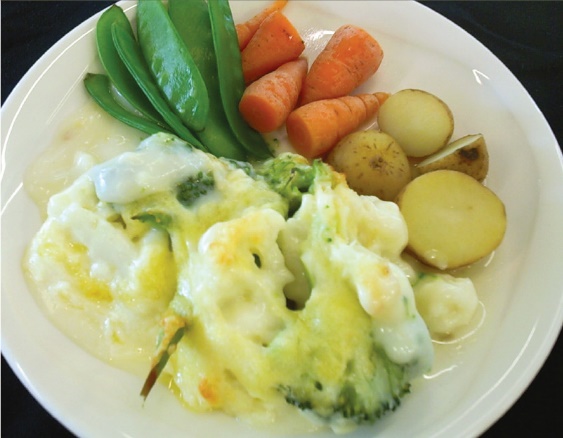 |
| Beef & Mushroom Pie   | Cheese, Onion & Potato Pie   |

**CONSORT 2010 Flow Diagram**

Assessed for eligibility (N/A; ineligible ppts automatically withdrawn)

Randomized (n= 2205)

**STUDY 3**

## Enrollment

## Allocation

Allocated to 50% meat-free options (n= 738)

♦ Received allocated intervention (n= 738)

Allocated to 75% meat-free options (n= 730)

♦ Received allocated intervention (n= 730)

Analysed (n= 729)
♦ Excluded from analysis (speeders: n= 1)

## Analysis

Analysed (n= 735)
♦ Excluded from analysis (speeders: n= 2)

Analysed (n= 737)
♦ Excluded from analysis (speeders: n= 1)

Allocated to 25% meat-free options (n= 737)

♦ Received allocated intervention (n= 737)
